# BDKRB1 Links Copy Number–Defined Genomic Instability to Inflammatory and Immunosuppressive Tumor Ecosystems in Ovarian Cancer: An Integrative Multiomics Analysis

**DOI:** 10.1155/ijog/2966274

**Published:** 2026-05-04

**Authors:** Dali Pu, Huagui Chen, Xia Wang, Shan He

**Affiliations:** ^1^ Sichuan Provincial Center for Gynecology and Breast Diseases (Gynecology), Affiliated Hospital of Southwest Medical University, Luzhou, China, ahswmu.cn; ^2^ The Affiliated Stomatological Hospital, Southwest Medical University, Luzhou, China, swmu.edu.cn

**Keywords:** BDKRB1, big data, drug sensitivity, immune microenvironment, multiomics integration, ovarian cancer, single-cell transcriptomics

## Abstract

**Background:**

Ovarian cancer is characterized by high mortality, extensive genomic instability driven by copy number alterations, and a highly immunosuppressive tumor microenvironment. Increasing evidence suggests that chronic inflammation and stromal–immune interactions contribute to tumor progression and therapeutic resistance. Bradykinin receptor B1 (BDKRB1), an inflammation‐inducible G protein–coupled receptor, has been implicated in tumor‐associated inflammatory signaling; however, its genomic determinants and immunological relevance in ovarian cancer remain poorly defined.

**Methods:**

We performed an integrative multiomics analysis of BDKRB1 using TCGA‐OV and multiple independent GEO cohorts. The analytical framework incorporated bulk transcriptomics, copy number variation profiling, single‐cell RNA sequencing, immune cell deconvolution, pathway enrichment analysis (GSEA, GSVA, and PROGENy), and pharmacogenomic modeling. Patients were dichotomized into BDKRB1‐high and BDKRB1‐low groups using cohort‐specific median expression to ensure cross‐dataset consistency. Associations with genomic instability, TME features, and drug response patterns were systematically evaluated. Quantitative real‐time polymerase chain reaction (qRT‐PCR) was further performed to validate BDKRB1 expression in ovarian cancer cell lines.

**Results:**

BDKRB1 was consistently overexpressed in ovarian cancer and associated with unfavorable clinical outcomes across multiple cohorts. Elevated BDKRB1 expression correlated with increased genomic instability, reflected by higher fractions of the genome altered, gained, and lost. Although copy number variation partially explained BDKRB1 upregulation, the modest correlation suggested additional regulatory mechanisms. Tumors with high BDKRB1 expression exhibited immunosuppressive microenvironmental features, including enrichment of cancer‐associated fibroblasts and reduced estimated CD8^+^ T‐cell infiltration, despite concurrent activation of inflammatory signaling pathways. Single‐cell transcriptomic analysis further identified fibroblasts as a major cellular source of BDKRB1 expression. Functional analyses indicated associations between BDKRB1 and inflammatory signaling, metabolic pathways, and oncogenic programs. Pharmacogenomic analyses suggested distinct drug sensitivity patterns in BDKRB1‐high tumors and identified fasudil as a potential candidate compound for reversing BDKRB1‐associated transcriptional signatures.

**Conclusion:**

This integrative analysis identifies BDKRB1 as a microenvironment‐associated marker linking genomic instability with inflammatory and immunosuppressive tumor ecosystems in ovarian cancer. Although the findings are primarily associative, they provide a systems‐level perspective on immune evasion mechanisms and highlight BDKRB1 as a potential biomarker for TME characterization and therapeutic hypothesis generation.

## 1. Introduction

Ovarian cancer is recognized as one of the most aggressive gynecologic malignancies, marked by high mortality rates due to late‐stage diagnosis and recurrence of the disease [1]. Treatment strategies have largely revolved around surgical debulking followed by platinum‐based chemotherapy; however, these interventions often yield limited long‐term efficacy as most patients develop resistance to therapy within a year of treatment initiation [2, 3]. Indeed, many patients with ovarian cancer exhibit recurrence due to platinum‐based chemotherapy resistance [4].

The development of disease‐specific targeted therapies has introduced some improvements in outcomes for certain patient subgroups, particularly with agents such as PARP inhibitors and anti‐angiogenic agents. For instance, the combination of olaparib and bevacizumab has been shown to extend progression‐free survival in certain populations of patients [5]. Despite these advancements, the overall survival (OS) benefits remain modest, underscoring the complex nature of ovarian cancer, characterized by its heterogeneity and dynamic tumor microenvironment (TME) that contribute to therapeutic resistance [6].

In light of these challenges, there is an urgent need to identify novel biomarkers that can predict clinical outcomes and potential therapeutic vulnerabilities in ovarian cancer. Understanding the molecular drivers that influence tumor progression, metastasis, and immune evasion could provide insight into new treatment strategies and facilitate improved patient stratification [3]. Investigations into genetic and pathobiological factors have demonstrated that specific epigenetic changes and signaling pathways, such as those involving MYPT1 and the Hippo pathway, are involved in resistance to platinum‐based therapies and could inform future therapeutic approaches [7, 8].

The bradykinin receptor B1 (BDKRB1) is an important candidate in cancer biology due to its role in various cellular processes linked to tumor progression and the TME. Unlike the B2 receptor, which is ubiquitously expressed, BDKRB1 is typically induced under pathological conditions such as inflammation or tissue injury, indicating its relevance in contexts including cancer [9]. Research suggests that BDKRB1 may play crucial roles in modulating angiogenesis, immune responses, and extracellular matrix (ECM) remodeling—factors integral to tumor growth and metastasis. For instance, studies have demonstrated that BDKRB1 signaling regulates aquaporin 4 expression in glioblastoma cells, implicating its influence on cell migration and invasion through a calcium‐dependent mechanism [10]. This underscores the potential for targeting BDKRB1 in strategies aimed at mitigating tumor aggressiveness. Furthermore, BDKRB1 has been associated with immune modulation in the TME. Activation of BDKRB1 has been linked to the polarization of tumor‐associated macrophages (TAMs) toward an M2‐like phenotype, which is known to drive immunosuppression and promote cancer progression [11]. Importantly, emerging evidence also suggests that BDKRB1 may exhibit context‐dependent associations across cancer types, potentially reflecting differences in dominant cellular sources and microenvironmental constraints. Moreover, there is evidence suggesting that BDKRB1 may function as a tumor suppressor gene, contributing to the maintenance of genomic stability through pathways involved in DNA repair and cell cycle checkpoint activation [12]. Together, these observations highlight that BDKRB1 biology may be multifaceted and ecosystem‐dependent rather than uniformly tumor cell–intrinsic.

Despite these advances, the precise mechanisms by which BDKRB1 influences ovarian cancer pathology, particularly its interaction with other signaling pathways and its effects on genomic integrity, remain inadequately understood. Here, we address this gap by providing an integrative, multicohort characterization of BDKRB1 that bridges genomic instability, immune–stromal organization, and pathway‐level activity in ovarian cancer. Specifically, we show that BDKRB1 is significantly overexpressed in ovarian tumors compared with normal tissue and is associated with adverse clinical outcomes across multiple cohorts. We further link BDKRB1 to copy number–defined genomic instability, quantified using genome‐wide CNA burden metrics (fraction of genome altered [FGA]/gained/lost), and demonstrate that the copy number–expression relationship is statistically significant yet modest, consistent with additional nongenetic regulatory inputs. At the microenvironment level, high BDKRB1 expression is associated with stromal remodeling and immune constraint, including cancer‐associated fibroblast (CAF) enrichment and reduced estimated CD8^+^ T‐cell abundance, while exhibiting an “inflamed but ineffective” configuration in which immune activation signatures coexist with immunosuppressive features. Single‐cell analyses localize BDKRB1 expression predominantly to fibroblast populations, supporting a stromal‐centered interpretation of bulk associations. Finally, we present exploratory pharmacogenomic and connectivity mapping analyses that nominate candidate therapeutic hypotheses for future validation. Collectively, this study reframes BDKRB1 as a stromal‐enriched, microenvironment‐associated marker linking copy number–defined genomic instability with inflammatory and immunosuppressive tumor ecosystems in ovarian cancer, providing a systems‐level framework for hypothesis generation rather than causal inference.

## 2. Methods

### 2.1. Data Acquisition and Processing

This study utilized uniformly processed ovarian serous cystadenocarcinoma (OV) transcriptomic data from The Cancer Genome Atlas (TCGA‐OV) cohort, accessed via the UCSC Xena Browser (https://xenabrowser.net/datapages/). Gene expression values were standardized to *Z*‐scores using *Z* = (*x* − *μ*)/*σ* to reduce inter‐sample scaling differences, with outliers (|*Z*| > 3) excluded to minimize technical artifacts. Authoritative clinical survival information (OS, disease‐specific survival [DSS], progression‐free interval [PFI], and disease‐free interval [DFI]) was sourced from the TCGA Pan‐Cancer Clinical Data Resource (TCGA‐CDR) published in the study by Liu et al. [13], the TCGA‐endorsed gold standard for outcome analyses. Independent validation cohorts GSE32063 and GSE73614 were obtained from the Gene Expression Omnibus (GEO). Unless otherwise specified, BDKRB1‐high and BDKRB1‐low groups were defined using the cohort‐specific median expression to facilitate cross‐dataset comparability.

### 2.2. GISTIC‐Based Copy Number Alteration Profiling

Genomic copy number variations (CNVs) were analyzed based on GISTIC scores derived from 427 tumor samples. Chromosomal‐level GISTIC scores were visualized using bar plots, with red bars indicating higher scores (copy number gains) and green bars denoting lower scores (copy number losses). To quantify total genomic alterations, each genomic segment was assigned a genomic distance calculated as half the distance between adjacent segment midpoints, or for terminal segments, the distance to chromosome ends. The FGA was defined as the proportion of autosomal genomic distance covered by altered segments relative to the whole‐genome distance. The fraction of genome gained (FGG) and fraction of genome lost (FGL) were similarly computed considering only gained or lost segments, respectively, following established methodology (genomic copy number alterations in clear cell renal carcinoma: associations with case characteristics and mechanisms of VHL gene inactivation).

Operational definition of genomic instability: In this study, “genomic instability” refers to genome‐wide CNA burden summarized by FGA, FGG, and FGL (length‐weighted fractions of the autosomal genome showing copy number alteration).

Analysis of variance (ANOVA) was employed to compare mean values across BDKRB1 expression–defined subgroups. When ANOVA revealed significant inter‐group differences (*p* < 0.05), TukeyHSD post hoc testing was performed to identify specific subgroup differences, reporting mean differences with 95% confidence intervals and adjusted *p* values. The association between CNV scores (GISTIC) and gene expression levels was assessed using scatter plots to visualize relationships, complemented by Spearman’s rank correlation coefficient to quantify monotonic associations independent of data distribution. Spearman’s *ρ* values range from −1 to 1, with extremes indicating strong correlations, and *p* values < 0.05 denoting statistical significance. Effect sizes were interpreted alongside significance to avoid overemphasis of modest correlations.

### 2.3. Immune Microenvironment Characterization

Immunomodulators (encompassing immunostimulators and immunosuppressors), chemokines, and human leukocyte antigens were obtained from the TISIDB database (http://cis.hku.hk/TISIDB/download.php). Differential expression of these immune molecules between BDKRB1‐high and BDKRB1‐low expression groups (stratified by median) was assessed using Wilcoxon rank‐sum tests, with heatmaps visualizing mean expression levels per gene across groups. For multigene panels, multiple testing was controlled using the Benjamini–Hochberg false discovery rate (FDR) where appropriate (see Statistical analysis).

The EaSIeR computational framework predicted antitumor immune responses from RNA‐seq data, evaluating five biomarkers: cytolytic activity (CYT), tertiary lymphoid structure (TLS), interferon‐γ (IFN‐γ) response, T‐cell inflamed signature, and chemokine activity [14]. These biomarkers were interpreted as gene expression–based immune activity signatures rather than direct estimates of immune cell abundance.

Patients were categorized into expression quartiles (Q1: highest 25%; Q4: lowest 25%) based on BDKRB1 levels. Following Thorsson et al.′s methodology (PMID: 29628290), mean biomarker scores per quartile were computed (excluding missing values) and visualized via hierarchical clustering heatmaps using the pheatmap R package. Quartile stratification was used here to visualize dose–response patterns, whereas median‐based dichotomization was used as the default grouping strategy elsewhere for comparability.

### 2.4. Immune Infiltration Assessment

To ensure data quality and consistency, immune infiltration profiles for all TCGA samples were acquired from the TIMER2.0 database [15] (http://timer.cistrome.org/). Multiple deconvolution algorithms comprehensively quantified diverse immune cell abundances and their correlations with BDKRB1 expression. These correlations were visualized via scatter plots to illustrate relationships between immune cell types and gene expression levels.

Samples were dichotomized into BDKRB1‐high and BDKRB1‐low groups based on median gene expression. Differences in algorithm‐inferred immune cell abundances between expression groups were evaluated using nonparametric Wilcoxon rank‐sum tests. Because deconvolution methods differ in reference signatures and modeling assumptions, immune infiltration results were interpreted based on directional concordance across algorithms rather than absolute estimates from any single method, and potential inter‐algorithm variability was acknowledged in interpretation.

Conceptual distinction of immune metrics: Deconvolution outputs were treated as estimated immune cell abundance/composition, whereas CYT/IFN‐γ/TIS/TLS and related scores were treated as gene expression–based immune activity signatures.

### 2.5. Functional Enrichment Characterization

Samples were dichotomized into BDKRB1‐high and BDKRB1‐low expression groups using the cohort‐specific median. Differential expression analysis was performed using the limma package in R, calculating log2‐fold changes (log2FC) and associated signed statistics to quantify expression differences. For preranked Gene Set Enrichment Analysis (GSEA), genes were ranked using a direction‐preserving statistic (e.g., signed log2FC or signed test statistic), rather than absolute values, to retain biological directionality.

GSEA was conducted using the clusterProfiler package across five databases: GO biological processes (GO‐BP), GO molecular functions (GO‐MF), GO cellular components (GO‐CC), Reactome pathways, and WikiPathways [16]. This computed normalized enrichment scores (NES) with significance testing and multiple hypothesis correction. Concurrently, GSVA (Gene Set Variation Analysis) with the z‐score algorithm quantified pathway activity for KEGG metabolic gene sets. GSVA was performed on normalized expression matrices without prior gene ranking, producing sample‐wise pathway activity scores and enabling assessment of pathway heterogeneity across individuals.

Complementary oncogenic pathway analysis was performed using the PROGENy algorithm (Version 1.10.0) implemented in the easier R package [14]. This method quantified activity scores for 14 signaling pathways: Androgen, EGFR, Estrogen, Hypoxia, JAK–STAT, MAPK, NF‐κB, p53, PI3K, TGF‐β, TNF‐α, Trail, VEGF, and Wnt. Pathway‐specific signatures were derived from perturbation experiments identifying genes responsive to pathway modulation, with activity scores inferred through linear regression modeling of affected genes. Importantly, PROGENy scores represent downstream transcriptional footprints of pathway signaling rather than direct upstream activation states (e.g., ligand levels or protein phosphorylation). To prevent circularity, 448 genes overlapping with immune response proxies were excluded prior to calculation (mean pan‐cancer Pearson correlation with original pathway activity: *r* = 0.99, *p* < 10^−16^). Pathway scores underwent *Z*‐score normalization (*Z* = (*x* − *μ*)/*σ*). Samples were stratified into BDKRB1‐high and BDKRB1‐low groups based on median gene expression. Differential pathway activity between groups was then evaluated to infer BDKRB1‐associated oncogenic mechanisms.

### 2.6. Cell‐Type‐Specific Mapping of BDKRB1 in Ovarian TME

Single‐cell transcriptomes were profiled using the TISCH database (Tumor Immune Single‐cell Hub) [17]. Cross‐tumor expression patterns of BDKRB1 were depicted in a heatmap, with Z‐score normalized expression values and inter‐sample relationships defined by Euclidean distance metrics. Hierarchical clustering applied the Ward algorithm to optimize cellular and transcriptional architecture. The TISCH2 analytical pipeline provided batch‐effect–corrected cell type annotations (e.g., T cells, B cells, and malignant populations). Cell type‐specific mean expression values for the target gene were computed and visualized via ggplot2‐generated bar plots.

Batch correction and annotation harmonization: Datasets derived from different platforms were processed under a unified workflow, and batch‐aware integration provided by the TISCH2 pipeline was used to mitigate platform‐related effects. Cell‐type labels were interpreted with reference to original study annotations and curated marker genes to support annotation consistency across datasets.

To mitigate scRNA‐seq technical limitations arising from sparse expression signals, the Nebulosa framework implemented weighted kernel density estimation [18]. This probabilistic approach reconstructs gene expression landscapes by modeling underlying transcriptional distributions.

Definition of BDKRB1‐positive cells: To delineate BDKRB1 expression within the ovarian cancer ecosystem, cells were dichotomized into BDKRB1‐positive and BDKRB1‐negative cohorts based on detectable (nonzero) transcript signal after normalization, recognizing that dropout‐related sparsity may lead to under‐detection of low‐abundance transcripts. Cellular subtype proportions within these expression‐defined compartments were quantified to identify principal contributors to BDKRB1 transcriptional output. This strategy supports a stromal‐centered interpretation when expression is enriched in fibroblast compartments.

### 2.7. Therapeutic Response Prediction

Chemotherapeutic outcome projections were generated using the pRRophetic R package [19]. This framework employs ridge regression models trained on GDSC1/GDSC2 reference datasets, correlating basal gene expression profiles with in vitro drug sensitivity to estimate half‐maximal inhibitory concentrations (IC50) across patient cohorts.

Spearman correlations quantified associations between BDKRB1 expression and pharmacodynamic metrics (IC50). Negative correlations indicated that elevated BDKRB1 transcript abundance coincided with reduced IC50 values, suggesting heightened therapeutic susceptibility. Conversely, positive correlations denoted BDKRB1‐associated resistance phenotypes. Because these analyses are derived from in vitro reference datasets with lineage heterogeneity, results were treated as exploratory and hypothesis‐generating, requiring disease‐specific validation.

To further identify compounds counteracting BDKRB1‐driven oncogenicity, a connectivity map (cMAP) screen implemented the XSum algorithm [20]. A 300‐gene signature (150 up/downregulated genes in BDKRB1‐high tumors) was cross‐referenced against 1288 compound‐induced transcriptional profiles. The analytical process followed the methodology outlined in previous publications [21]. The 300‐gene signature size was selected as a pragmatic balance between robustness and noise control for connectivity mapping; alternative signature sizes were not systematically explored and are therefore acknowledged as a limitation. Compounds yielding minimal similarity scores were prioritized as candidates for reversing BDKRB1‐mediated tumorigenic pathways, and fasudil is reported as a representative top‐ranked candidate based on connectivity‐based prioritization rather than a uniquely definitive hit.

### 2.8. Quantitative Real‐Time polymerase chain reaction (qRT‐PCR) Analysis

qRT‐PCR was performed using the human ovary cell line IOSE80 and ovarian cancer cell lines (A2780 and SKOV3) were obtained from the cell repository of the Central Laboratory of the Affiliated Hospital of Southwest Medical University. They were cultured at 37°C with 5% CO2. Total RNA was refined using the RNA extraction kit (Promega, LS1040) according to the manufacturer’s instructions. cDNAs were composed by reverse transcription. Q‐PCR was carried out using the SYBR Green Master kit (Roche). The primers used were as follows: for BDKRB1 (PrimerBank ID 347300338c1, amplicon size 158 bp), forward 5′‐AAT​GCT​ACG​GCC​TGT​GAC​AAT‐3′ and reverse 5′‐ATT​TCT​GCC​ACG​TTC​AGT​TGC‐3′; for GAPDH, forward 5′‐GGA​AGC​TTG​TCA​TCA​ATG​GAA​ATC‐3′ and reverse 5′‐TGA​TGA​CCC​TTT​TGG​CTC​CC‐3′. The mRNA expression level of the BDKRB1 gene was normalized to GAPDH.

### 2.9. Statistical Analysis

Data analyses were performed in R and Python. Two‐group comparisons used the Wilcoxon rank‐sum test, and associations were assessed using Spearman’s rank correlation. Unless otherwise specified, statistical significance was defined as two‐sided *p* < 0.05. For analyses involving multiple comparisons (e.g., multigene immune panels and gene set/pathway testing), *p* values were adjusted using the Benjamini–Hochberg FDR; analyses based on limited predefined hypotheses were reported using nominal *p* values and interpreted as exploratory, with the multiple‐testing approach indicated in the relevant Methods subsection and figure legend. For Cox regression, proportional hazards assumptions were assessed using Schoenfeld residual–based diagnostics; models were considered appropriate when no meaningful violations were detected.

## 3. Result

### 3.1. BDKRB1 Overexpression is Associated With Poor Prognosis in Ovarian Cancer

Differential expression analysis integrating TCGA‐OV tumors with GTEx normal controls demonstrated significant upregulation of BDKRB1 in ovarian cancer tissues (*p* = 0.029) (Figure 1(a)). An exploratory diagnostic model constructed from this expression difference showed acceptable calibration, with no significant deviation in the Hosmer–Lemeshow test (*p* = 0.441), and is presented here as a proof‐of‐concept rather than a clinically deployable tool (Figure 1(b)). Experimental validation using qRT‐PCR confirmed elevated BDKRB1 expression in ovarian cancer cell lines (A2780 and SKOV3) compared with the nonmalignant IOSE80 line (Figure 1(c)), while immunohistochemical data from the Human Protein Atlas further supported increased BDKRB1 protein expression in tumor tissues relative to normal ovarian epithelium (Figure 1(d)).

FIGURE 1BDKRB1 overexpression in ovarian cancer and its prognostic relevance. (a) Differential BDKRB1 mRNA expression between TCGA‐OV tumors and GTEx normal ovarian tissues. (b) Calibration curve of an exploratory BDKRB1‐based diagnostic model, with the Hosmer–Lemeshow test assessing model fit. (c) qRT‐PCR validation of BDKRB1 upregulation in ovarian cancer cell lines (A2780 and SKOV3) relative to the nonmalignant IOSE80 line. (d) Immunohistochemical evidence of elevated BDKRB1 protein expression in ovarian carcinoma compared with normal ovarian epithelium (human protein atlas). (e) Summary of prognostic associations across survival endpoints (OS, DSS, PFI, and DFI) based on Cox and log‐rank analyses. (f–h) Kaplan–Meier survival curves for OS, DSS, and PFI in TCGA‐OV cohorts stratified by BDKRB1 expression. (i–k) External validation of OS associations in GEO cohorts (GSE32063 and GSE73614) using median‐based stratification, with outcome‐optimized cutoffs shown where indicated for visualization.

**Figure:**
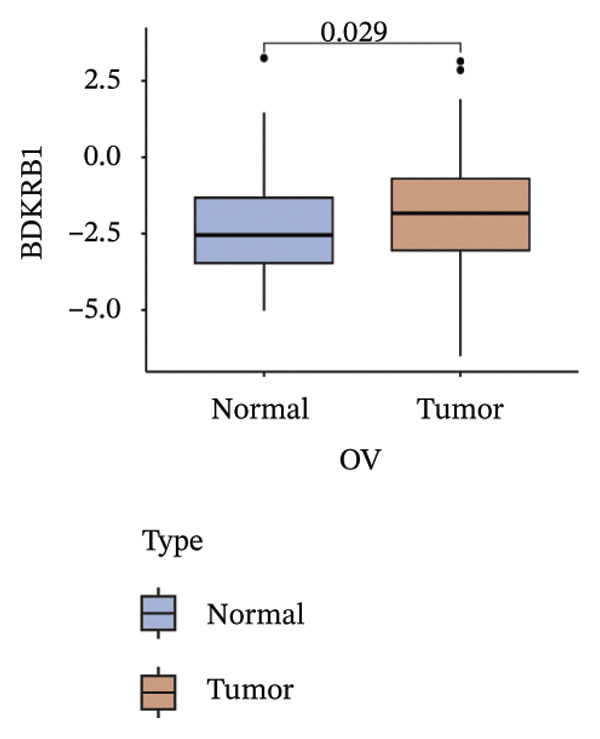
(a)

**Figure:**
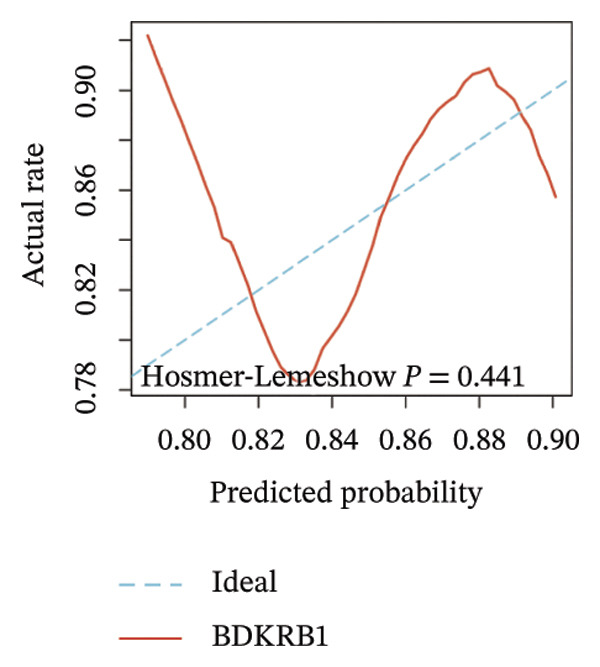
(b)

**Figure:**
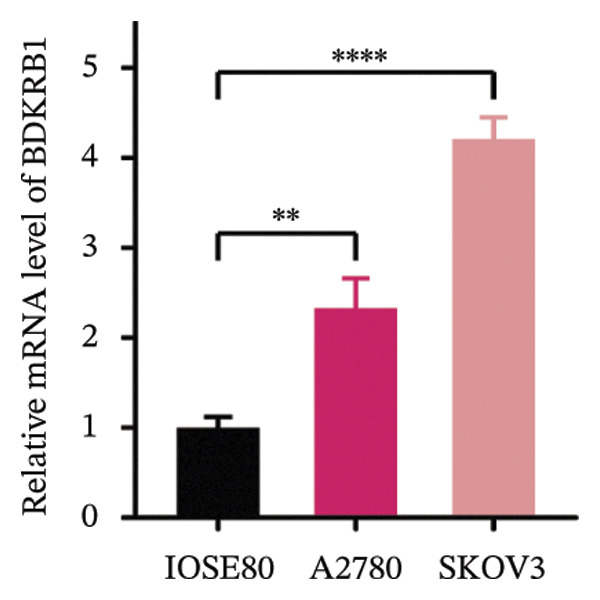
(c)

**Figure:**
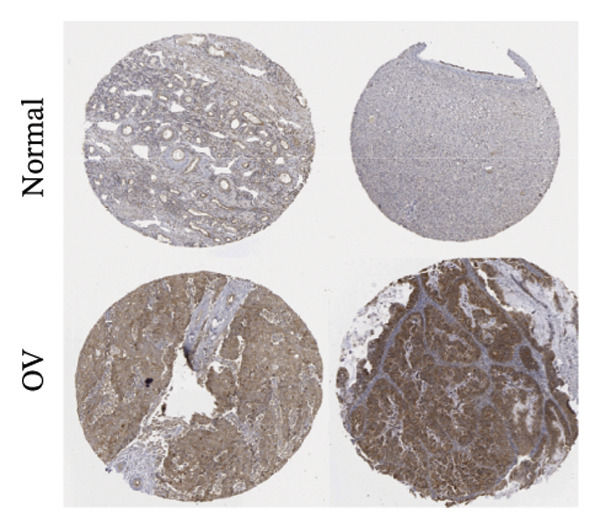
(d)

**Figure:**
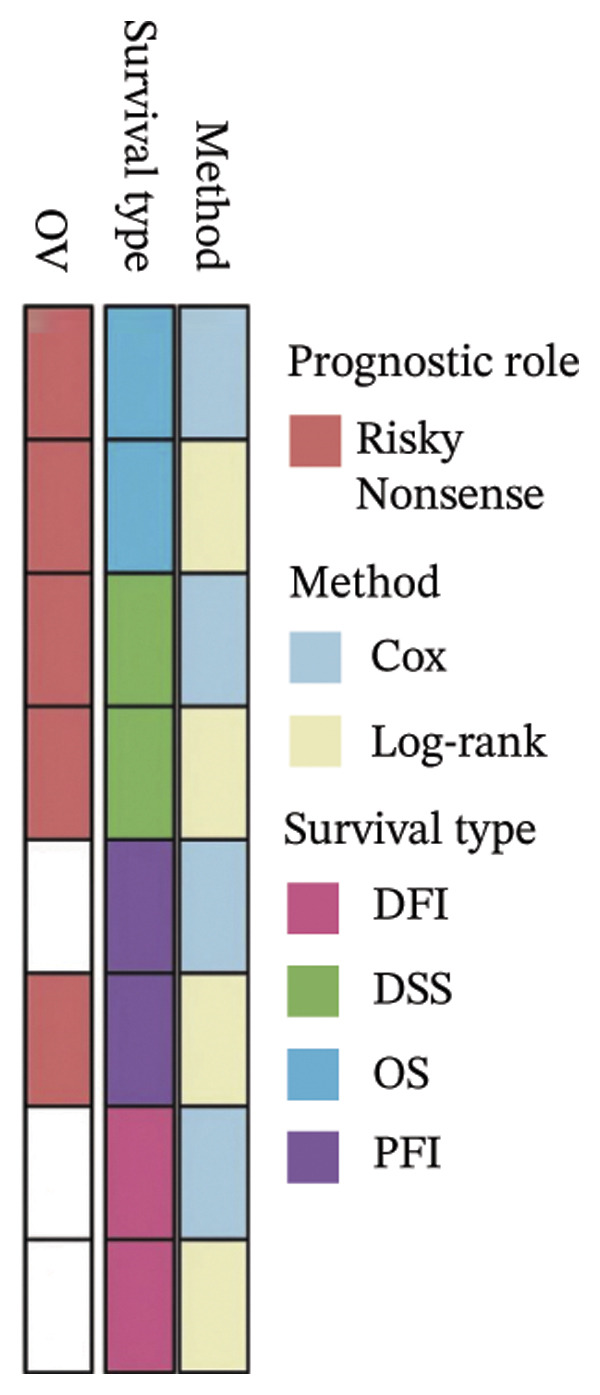
(e)

**Figure:**
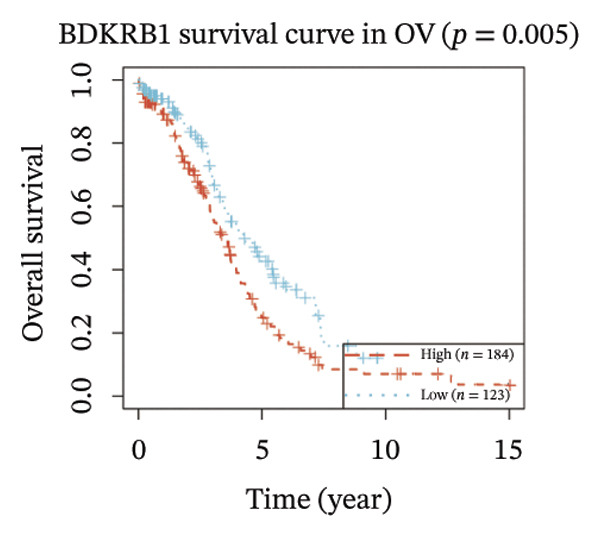
(f)

**Figure:**
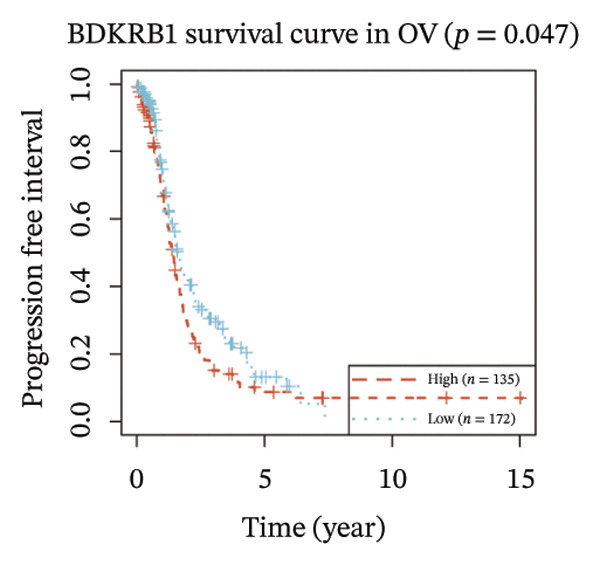
(g)

**Figure:**
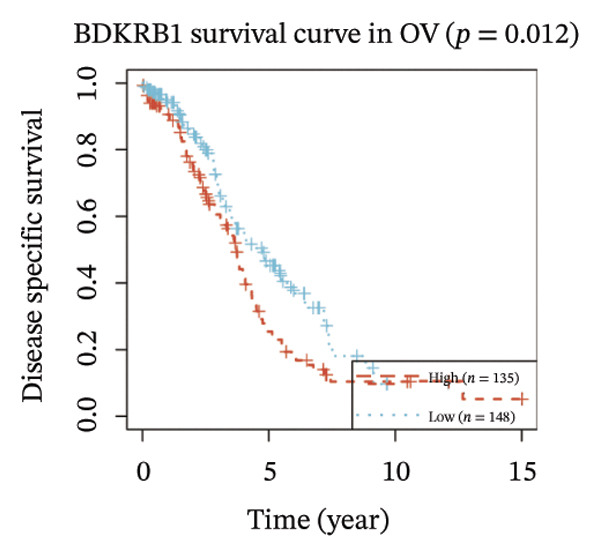
(h)

**Figure:**
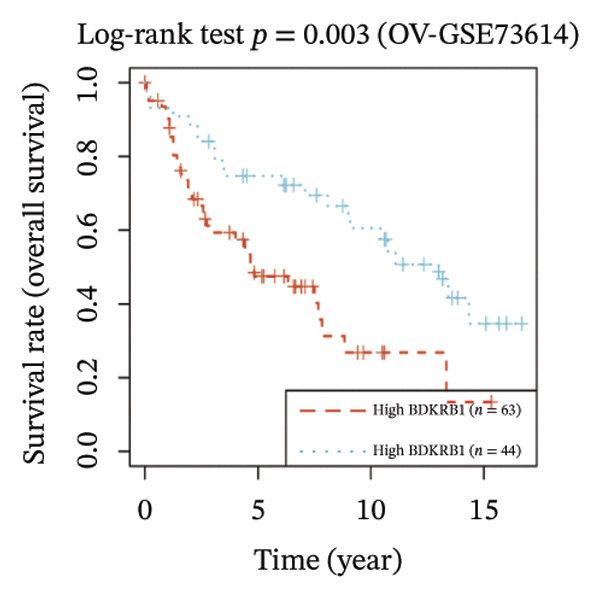
(i)

**Figure:**
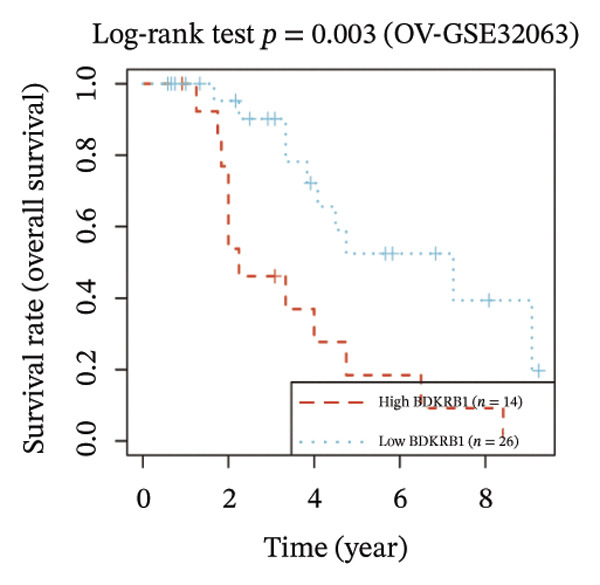
(j)

**Figure:**
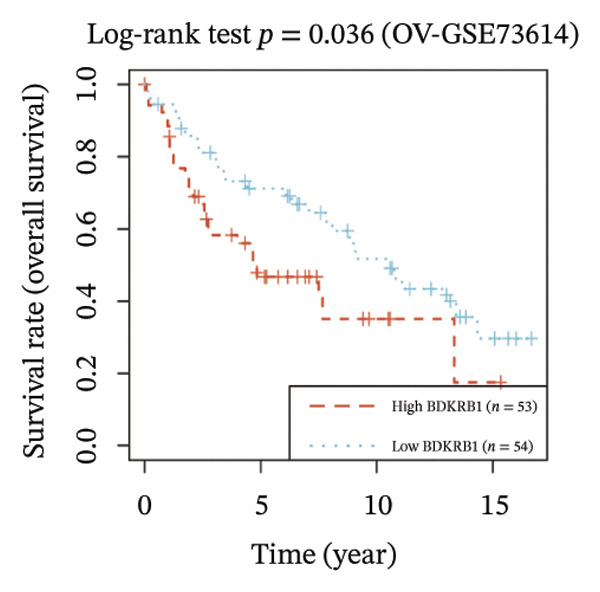
(k)

Prognostic analyses revealed endpoint‐specific associations with high BDKRB1 expression was consistently associated with worse OS (Cox *p* < 0.001, log‐rank *p* = 0.005) and DSS (Cox *p* < 0.001, log‐rank *p* = 0.012). In contrast, PFI showed partial discordance, with significance observed in log‐rank testing (*p* = 0.047) but not in Cox models, and DFI showed no significant association ((Figure 1(e))). These differences may reflect the distinct biological processes captured by long‐term survival endpoints versus earlier recurrence dynamics. Kaplan–Meier analyses further demonstrated that elevated BDKRB1 expression was associated with poorer OS, DSS, and PFI in TCGA‐OV cohorts (Figures 1(f), 1(g), and 1(h)). Using cohort‐specific median‐based dichotomization as the primary stratification strategy, these findings were validated in independent GEO datasets, where high BDKRB1 expression consistently predicted inferior OS in both GSE32063 and GSE73614, with additional outcome‐optimized cutoffs shown for visualization (Figures 1(i), 1(j), and 1(k)). Collectively, multicohort evidence supports BDKRB1 overexpression as a robust adverse prognostic correlate in ovarian cancer.

### 3.2. BDKRB1‐Associated Genomic Instability and Therapeutic Implications in OV

In TCGA‐OV ovarian serous cystadenocarcinoma samples, recurrent somatic copy number alteration (SCNA) patterns were observed across multiple chromosomes, with several regions showing recurrent gains (e.g., chr3/chr8/chr19) and others showing recurrent losses (e.g., chr5/chr22) as summarized by GISTIC scores (Figure 2(a)). At the gene level, BDKRB1 copy number (GISTIC2 scores) showed a statistically significant but modest positive association with BDKRB1 mRNA expression (Spearman’s *ρ* = 0.21, *p* = 0.000222) (Figure 2(b)), suggesting that copy number dosage may partially contribute to transcriptional variability while nongenetic regulatory mechanisms are also likely involved. Consistent with a genome‐wide instability phenotype, stratification by BDKRB1 expression quartiles revealed that the highest‐expression group exhibited significantly increased CNA burden metrics—FGA, FGG, and FGL—relative to lower‐expression groups (Tukey HSD, *p* < 0.05) (Figure 2(c)). Together, these results indicate that BDKRB1‐high tumors tend to co‐occur with elevated copy number–defined genomic instability, a feature that may reflect greater chromosomal imbalance and tumor heterogeneity. Therapeutically, these findings are hypothesis‐generating and suggest that BDKRB1‐high/instability‐high tumors may warrant future investigation in the context of genome instability–targeted vulnerabilities, rather than constituting evidence for a specific clinical strategy.

FIGURE 2BDKRB1‐associated copy number alterations and copy number–defined genomic instability in ovarian cancer. (a) Genome‐wide recurrent somatic copy number alteration patterns in TCGA‐OV summarized by GISTIC scores. (b) Association between BDKRB1 copy number (GISTIC2 scores) and BDKRB1 mRNA expression in TCGA‐OV. (c) Copy number burden metrics—fraction of genome altered (FGA), fraction of genome gained (FGG), and fraction of genome lost (FGL)—across BDKRB1 expression quartiles (Tukey HSD, *p* < 0.05).

**Figure:**
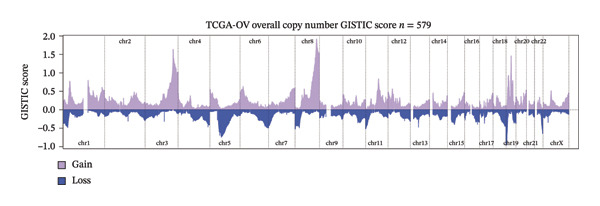
(a)

**Figure:**
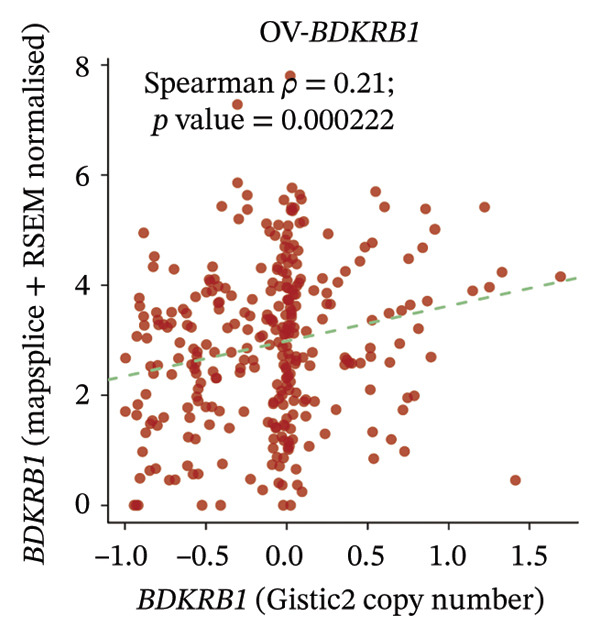
(b)

**Figure:**
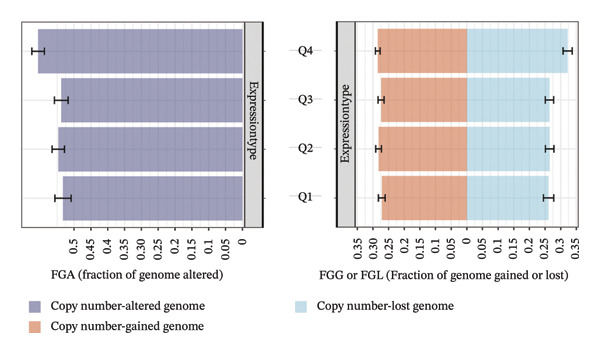
(c)

### 3.3. BDKRB1 Overexpression is Associated With an Inflamed but Functionally Constrained Immunogenomic Niche

Integrative immunogenomic analyses revealed that elevated BDKRB1 expression was associated with broad remodeling of immune‐related transcriptional networks in ovarian cancer. At the gene level, BDKRB1‐high tumors exhibited increased expression of several immunoregulatory mediators, including TGFB1, ADORA2A, and myeloid‐associated markers (e.g., CSF1R), alongside enhanced expression of antigen‐presentation molecules such as HLA‐DRA and HLA‐B. Chemokine signaling displayed mixed polarization, with upregulation of inflammatory chemokines (e.g., CCL5, CXCL2, and CXCL13) but relative suppression of selected lymphocyte‐recruiting signals (e.g., CCL19 and XCL2), indicating non‐uniform immune network remodeling (Figure 3(a)).

FIGURE 3Immunogenomic features associated with elevated BDKRB1 expression in ovarian cancer. (a) Heatmap showing differential expression of immune‐related genes between BDKRB1‐high and BDKRB1‐low tumors. (b–f) Comparisons of immune activity signatures (CYT, IFN‐γ response, T cell–inflamed signature, TLS, and chemokine signaling) between BDKRB1 expression groups. (g) Row‐standardized heatmap of immune and genome‐associated metrics across BDKRB1 expression quartiles, illustrating graded immunogenomic transitions.

**Figure:**
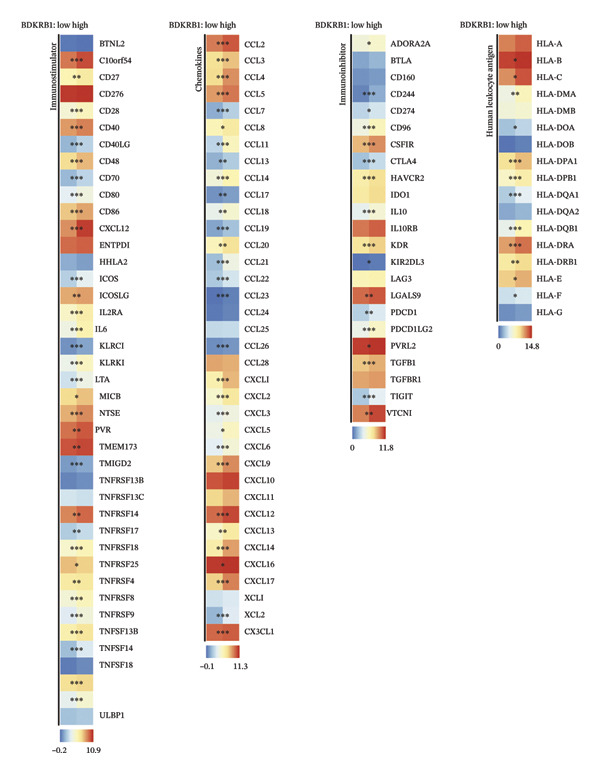
(a)

**Figure:**
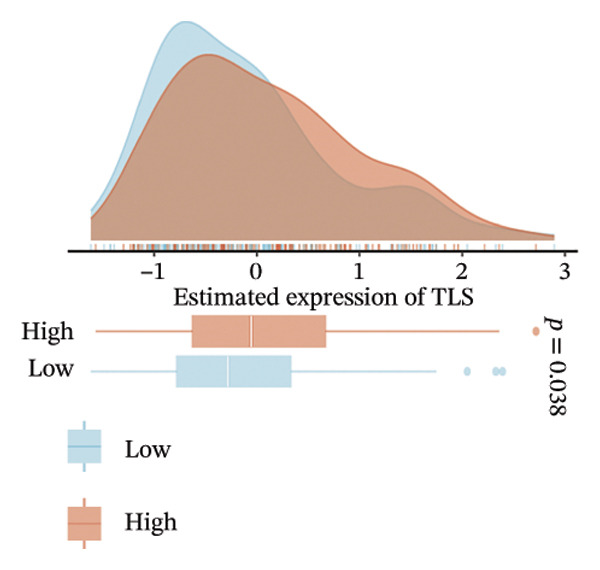
(b)

**Figure:**
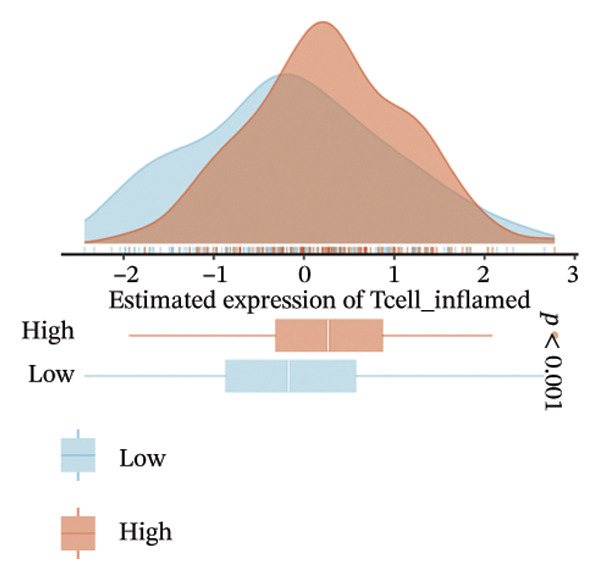
(c)

**Figure:**
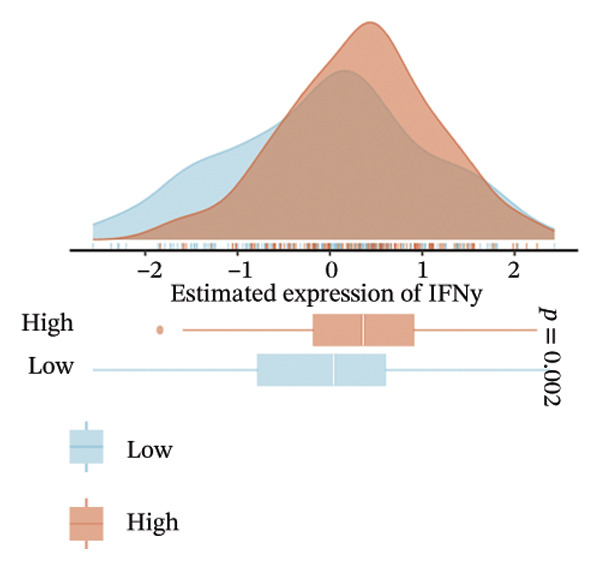
(d)

**Figure:**
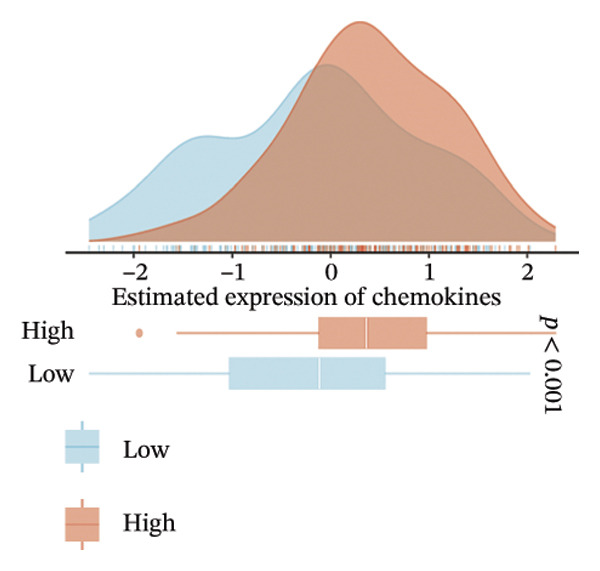
(e)

**Figure:**
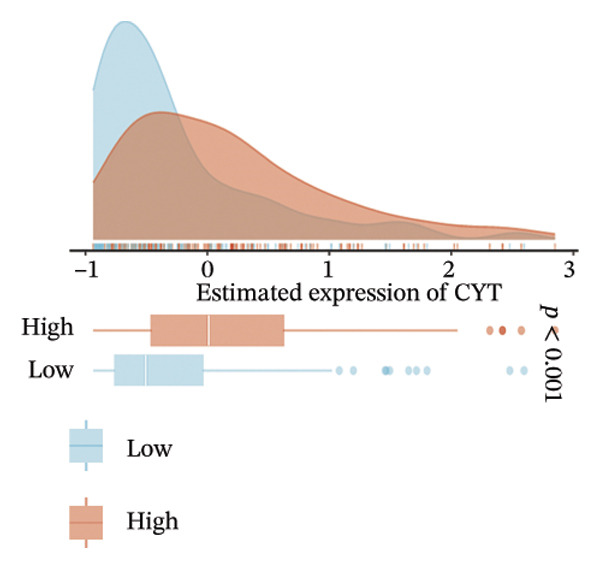
(f)

**Figure:**
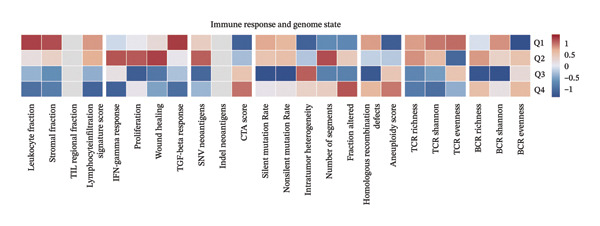
(g)

Despite enrichment of immunoregulatory and stromal‐associated signals, transcriptomic immune activity metrics were paradoxically elevated in BDKRB1‐high tumors. Wilcoxon rank‐sum comparisons demonstrated higher CYT (*p* < 0.001), IFN‐γ response (*p* = 0.002), T cell–inflamed signature (*p* < 0.001), TLS score (*p* = 0.038), and chemokine activity (Figures 3(b), 3(c), 3(d), 3(e), and 3(f)). This coexistence of immune activation signatures with immunoregulatory features supports an “inflamed but ineffective” immune configuration, in which inflammatory signaling is present but may be functionally constrained by stromal remodeling or suppressive microenvironmental circuits rather than reflecting uniformly productive antitumor immunity.

Stratification by BDKRB1 expression quartiles further highlighted graded immunogenomic transitions. Row‐standardized heatmap analysis across quartiles revealed coordinated variation in lymphocyte activation signatures, IFN‐γ signaling, TGF‐β response, and genome instability–related features such as aneuploidy and neoantigen burden (Figure 3(g)). These patterns suggest that BDKRB1 expression marks a continuum of tumor ecosystem states characterized by concurrent immune activation signals and structural immune constraint, consistent with a stromal‐influenced tumor–immune interface rather than a purely tumor cell–intrinsic immune phenotype.

### 3.4. BDKRB1 Expression is Associated With a Stromal‐Enriched and CD8^+^ T Cell–Constrained Microenvironment in OV

To estimate the cellular composition of the TME, we analyzed TCGA‐OV samples using multiple immune deconvolution frameworks (EPIC, TIMER, xCell, CIBERSORT‐ABS, quanTIseq, and MCP‐counter). Across methods, BDKRB1 expression showed a consistent positive association with CAF abundance (e.g., EPIC and MCP‐counter), together with higher stromal‐related scores (xCell), indicating a stromal‐enriched ecosystem in BDKRB1‐high tumors (Figure 4(a)). In contrast, BDKRB1 expression was inversely associated with estimated CD8^+^ T cell abundance in selected algorithms (e.g., EPIC), suggesting a CD8^+^ T cell–constrained configuration. When samples were dichotomized by the cohort‐specific median into BDKRB1‐high versus BDKRB1‐low groups, Wilcoxon rank‐sum comparisons similarly demonstrated increased inferred CAF/stromal signals and reduced inferred CD8^+^ T cell abundance in the BDKRB1‐high group across multiple deconvolution methods (Figure 4(b)). Taken together, and interpreted in light of inter‐algorithm variability, these concordant directional patterns support an association between elevated BDKRB1 expression and a microenvironment operationally characterized by stromal expansion and reduced cytotoxic T cell presence, consistent with a functionally constrained antitumor immune context.

FIGURE 4Immune cell abundance estimates associated with BDKRB1 expression in ovarian cancer. (a) Associations between BDKRB1 expression and deconvolution‐inferred cell abundances across multiple algorithms (EPIC, TIMER, xCell, CIBERSORT‐ABS, quanTIseq, and MCP‐counter). (b) Differences in inferred CAF/stromal signals and CD8^+^ T cell abundance between BDKRB1‐high and BDKRB1‐low tumors (median stratification; Wilcoxon rank‐sum test).

**Figure:**
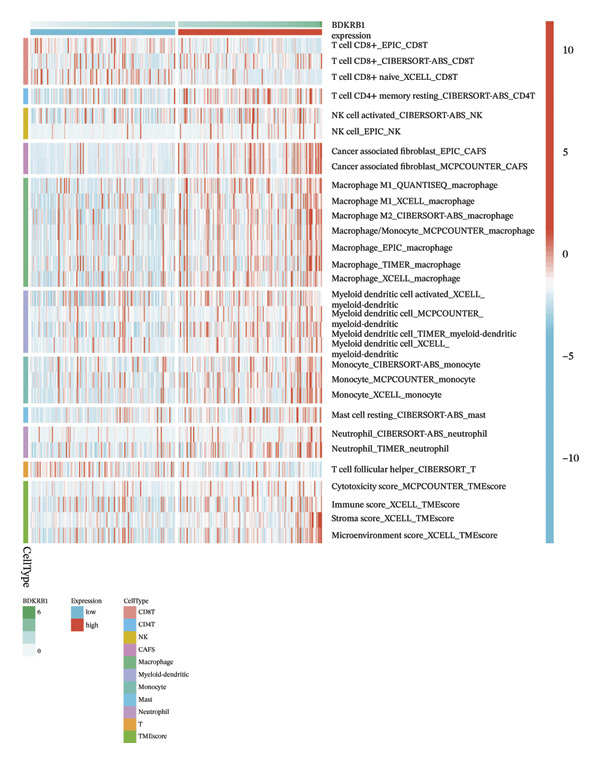
(a)

**Figure:**
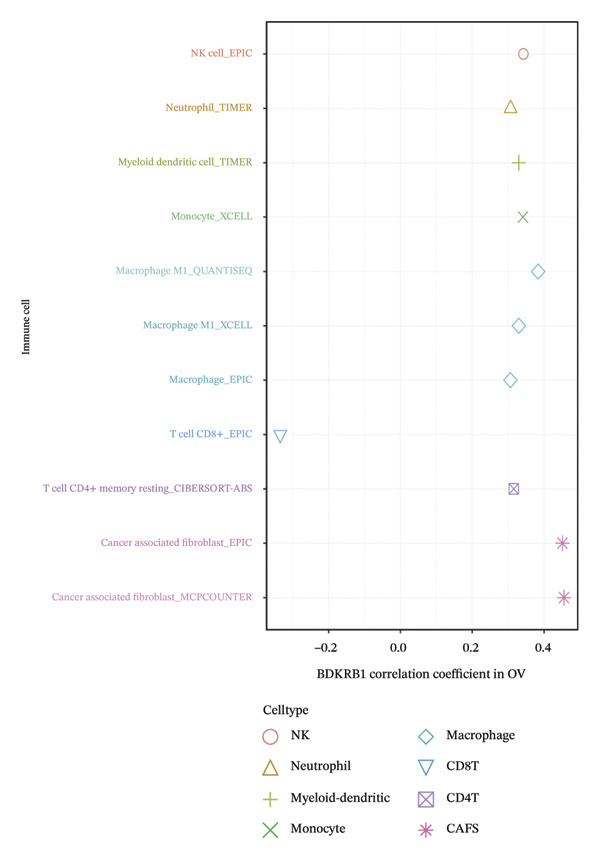
(b)

### 3.5. BDKRB1 Expression is Associated With Metabolic Remodeling and Broad Transcriptional Pathway Activity in Ovarian Cancer

Metabolic reprogramming was significantly associated with BDKRB1 expression levels. Notably, the BDKRB1 high‐expression group exhibited upregulation of multiple biosynthetic pathways, including glycosphingolipid biosynthesis (globo/isoglobo, lacto/neolacto, and ganglio series), glycosaminoglycan biosynthesis (chondroitin/dermatan sulfate and keratan sulfate), mucin type O‐glycan biosynthesis, steroid biosynthesis, and amino acid biosynthesis (phenylalanine/tyrosine/tryptophan and valine/leucine/isoleucine). Conversely, butanoate metabolism, mannose type O‐glycan biosynthesis, biotin metabolism, and lipoic acid metabolism were suppressed in high expressers, indicating that BDKRB1 overexpression promotes glycan/lipid biosynthesis while repressing cofactor and short‐chain fatty acid metabolism (Figure 5(a)).

FIGURE 5Metabolic and pathway‐level features associated with BDKRB1 expression in ovarian cancer. (a) GSVA‐derived activity differences in KEGG metabolic gene sets between BDKRB1‐high and BDKRB1‐low tumors. (b) Preranked GSEA showing enrichment of immune/inflammatory programs in BDKRB1‐high tumors and cilium‐related programs in BDKRB1‐low tumors. (c) Correlation analysis linking BDKRB1 expression with pathway signatures related to DNA damage response and EMT. (d) PROGENy‐inferred pathway activity scores across oncogenic signaling programs comparing BDKRB1‐high versus BDKRB1‐low tumors (Wilcoxon rank‐sum test; *p* values as indicated).

**Figure:**
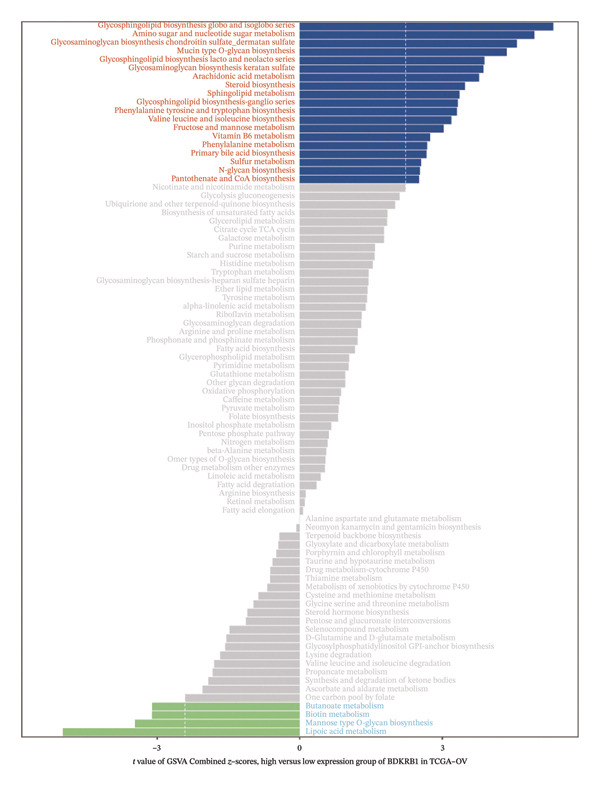
(a)

**Figure:**
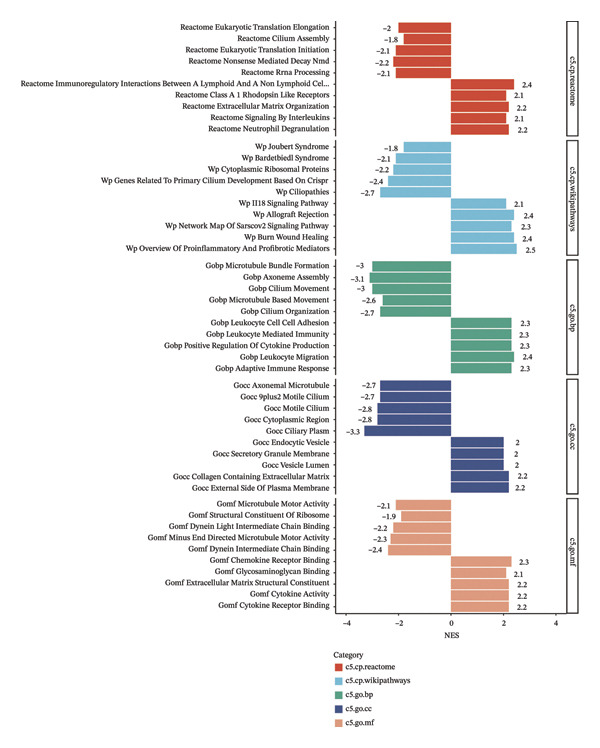
(b)

**Figure:**
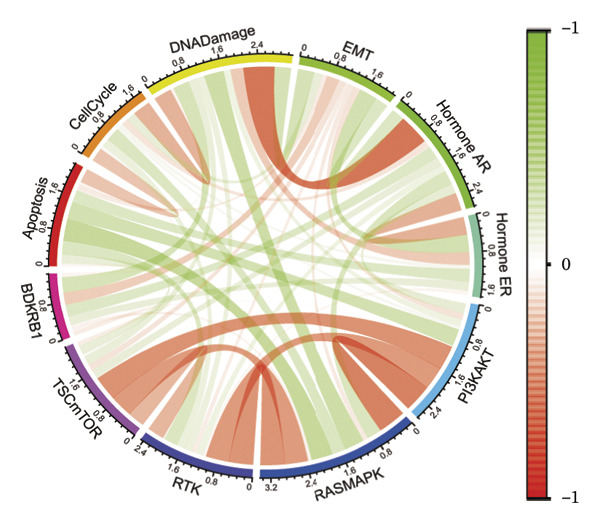
(c)

**Figure:**
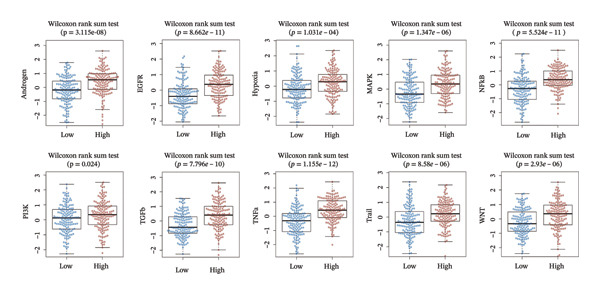
(d)

Consistent with immune remodeling observed elsewhere, preranked GSEA further identified enrichment of inflammatory and immune‐related programs in BDKRB1‐high tumors (e.g., Reactome “Neutrophil Degranulation,” “Signaling by Interleukins,” and “Immunoregulatory Interactions,” together with GO‐BP terms related to adaptive immune response and leukocyte migration), whereas cilium‐related processes (e.g., Reactome “Cilium Assembly” and GO categories related to cilium organization) were enriched in the BDKRB1‐low group (Figure 5(b)). In addition, correlation‐based analyses linked BDKRB1 expression to differential pathway states, including inverse associations with DNA damage–related signatures and positive associations with epithelial–mesenchymal transition (EMT) activity (Figure 5(c)). Finally, PROGENy‐based pathway activity inference (interpreted as downstream transcriptional footprints rather than direct upstream activation) demonstrated broadly higher signaling activity in the BDKRB1‐high group across multiple oncogenic pathways, including EGFR, MAPK, NF‐κB, TNFα, hypoxia, TGF‐β, WNT, TRAIL, and PI3K (all nominal *p* values as shown) (Figure 5(d)). Together, these multilayer pathway readouts support that elevated BDKRB1 expression is associated with a coordinated tumor ecosystem state characterized by enhanced biosynthetic metabolism, inflammatory signaling, and broad transcriptional pathway activity while remaining descriptive and not implying direct causal regulation by BDKRB1.

### 3.6. Single‐Cell Analyses Localize BDKRB1 Expression Predominantly to Fibroblast Compartments Across Ovarian Cancer Cohorts

Single‐cell transcriptomic analysis across four ovarian cancer cohorts (EMTAB8107, GSE130000, GSE147082, and GSE151214) identified fibroblasts as the predominant expressors of BDKRB1, with significantly elevated mean expression levels in this cell type compared to all others (Figures 6(a), 6(b), 6(c), and 6(d)). Specifically in the EMTAB8107 cohort, fibroblasts exhibited robustly elevated BDKRB1 expression over malignant, immune (CD8T, B), and stromal cells (endothelial and myofibroblasts). This pattern was reinforced in GSE130000 (*p* < 0.001), where fibroblasts demonstrated the highest BDKRB1 expression, surpassing CD8Tex, malignant, and myofibroblast populations. Consistent findings emerged in GSE147082 (*p* < 0.001), with fibroblasts showing markedly higher expression than CD4Tconv, endothelial, and malignant cells (≤ 0.01), and similarly in GSE151214 (*p* < 0.001), where fibroblasts significantly outperformed epithelial, immune (CD4T/CD8T), and other stromal components.

FIGURE 6Fibroblast‐enriched cellular origin of BDKRB1 expression across ovarian cancer scRNA‐seq cohorts. (a–d) BDKRB1 expression across annotated cell types in EMTAB8107, GSE130000, GSE147082, and GSE151214, showing consistent enrichment of BDKRB1 in fibroblast compartments relative to malignant and immune populations.

**Figure:**
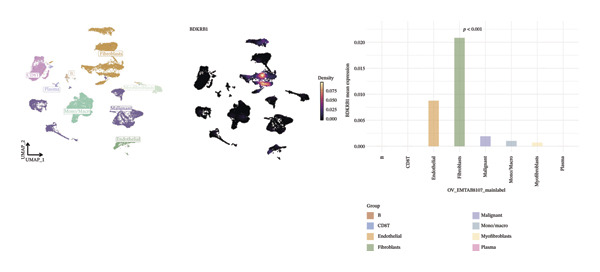
(a)

**Figure:**
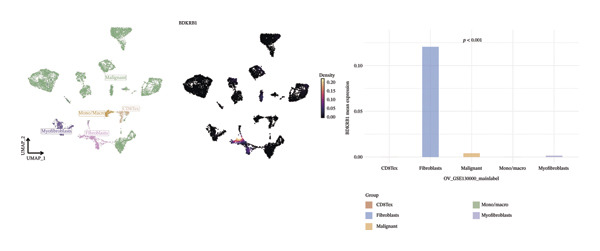
(b)

**Figure:**
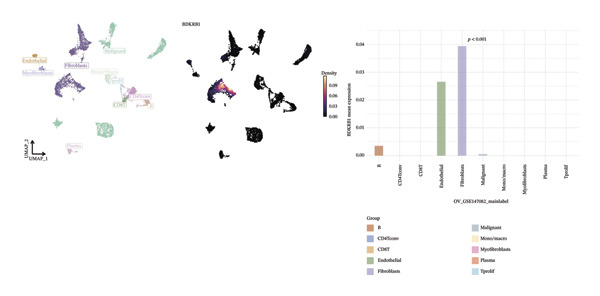
(c)

**Figure:**
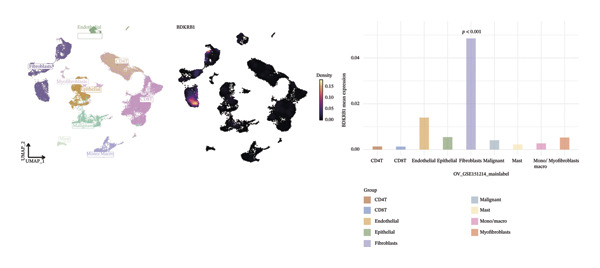
(d)

Consistent with these expression patterns, analysis of BDKRB1‐positive cells further showed that the BDKRB1‐detectable compartment was overwhelmingly composed of fibroblasts across cohorts, with fibroblasts accounting for the majority of BDKRB1‐positive cells (e.g., 95.6% in GSE147082, 68.2% in GSE130000, and 82.9% in EMTAB8107; cohort‐specific proportions as shown) (Figures 7(a), 7(b), 7(c), and 7(d)). Together, these single‐cell results indicate that BDKRB1 expression in ovarian cancer is predominantly stromal and fibroblast‐centered, providing essential context for interpreting bulk transcriptomic associations as reflecting tumor ecosystem organization rather than tumor cell–intrinsic expression.

FIGURE 7Fibroblasts dominate the BDKRB1‐positive compartment in ovarian cancer scRNA‐seq cohorts. (a–d) Cell‐type composition of BDKRB1‐positive cells in GSE147082, GSE151214, GSE130000, and EMTAB8107, showing that fibroblast subsets constitute the majority of BDKRB1‐detectable cells across datasets.

**Figure:**
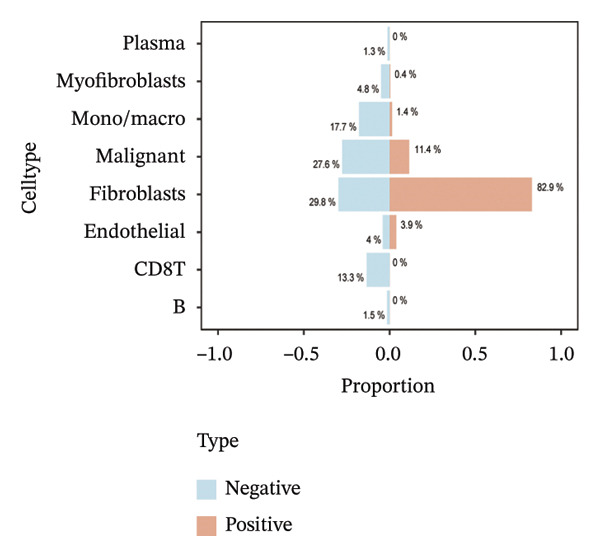
(a)

**Figure:**
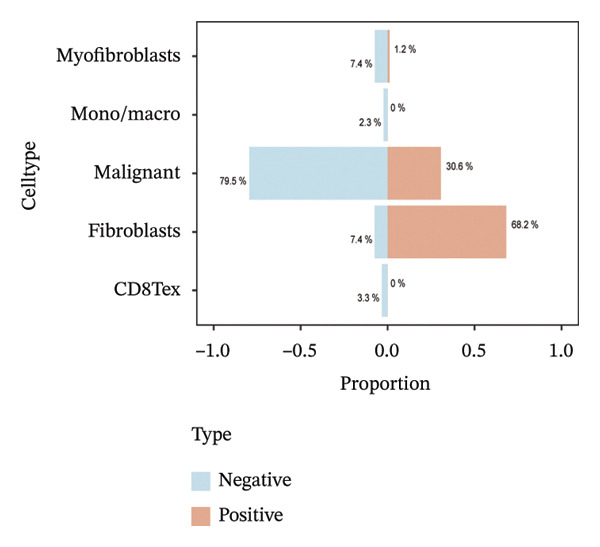
(b)

**Figure:**
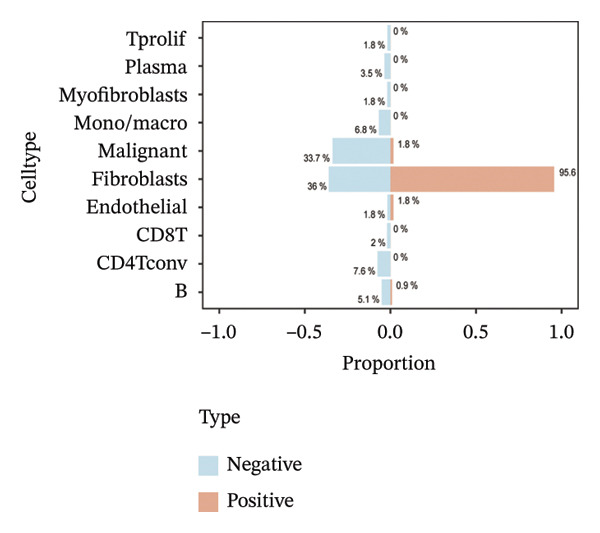
(c)

**Figure:**
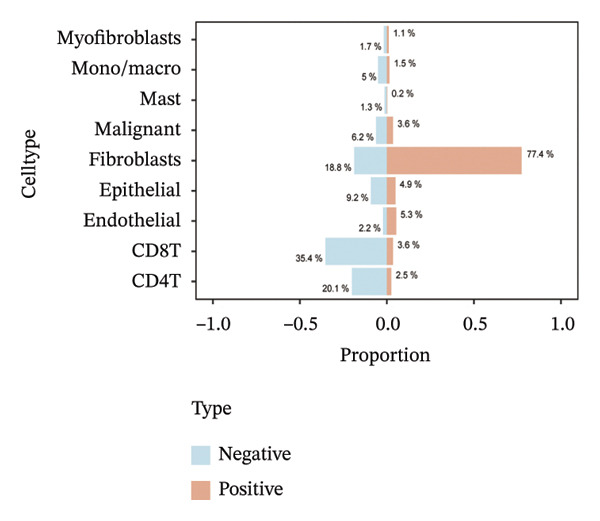
(d)

### 3.7. BDKRB1 Expression is Associated With Exploratory Pharmacogenomic Sensitivity Patterns and Connectivity‐Based Candidate Prioritization

Using pharmacogenomic reference datasets (GDSC1 and GDSC2), Spearman correlation analyses identified significant negative associations between BDKRB1 expression and predicted drug response metrics for selected compounds, including tanespimycin (HSP90 inhibitor) in GDSC1 and dasatinib (multikinase inhibitor) in GDSC2 (Figures 8(a) and 8(b)), consistent with lower estimated IC50 values at higher BDKRB1 expression. Because these inferences are derived from in vitro cell‐line resources with lineage heterogeneity, we interpret them as exploratory and hypothesis‐generating rather than clinically actionable evidence. To contextualize candidate compounds within broader biological programs, crosslink mapping of a curated 58‐agent library illustrated coverage of major cancer‐associated signaling and process modules, including cell cycle control, apoptosis, DNA replication/repair, and genome integrity–related pathways, alongside canonical signaling axes such as ERK/MAPK, PI3K/mTOR, RTK/WNT, chromatin regulation, and metabolic processes (Figure 8(c)).

FIGURE 8Exploratory pharmacogenomic associations and connectivity‐based candidate compounds linked to BDKRB1. (a, b) Spearman correlations between BDKRB1 expression and in vitro drug response metrics in GDSC1 (tanespimycin) and GDSC2 (dasatinib). (c) Gene–drug–pathway network summarizing major signaling/process modules covered by the curated compound set. (d) Connectivity map (cMAP) analysis using the XSum algorithm to prioritize compounds predicted to inversely relate to the BDKRB1‐associated 300‐gene signature, highlighting fasudil as a representative top‐ranked candidate.

**Figure:**
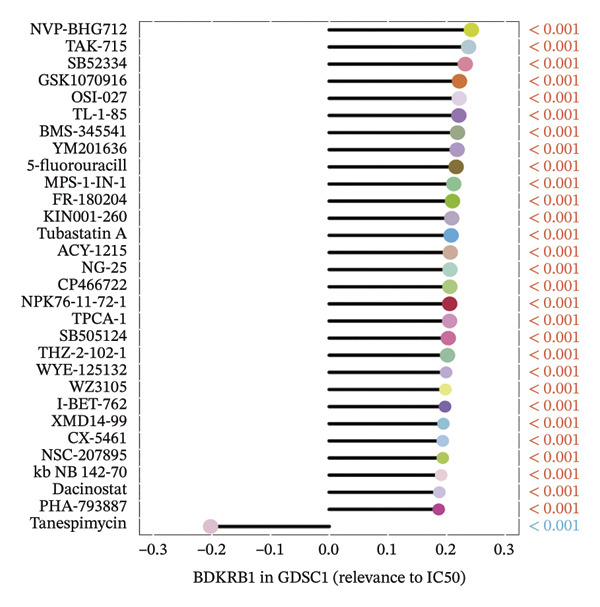
(a)

**Figure:**
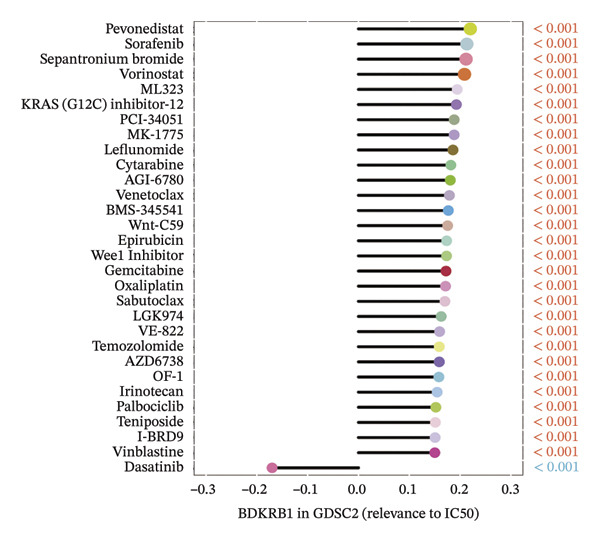
(b)

**Figure:**
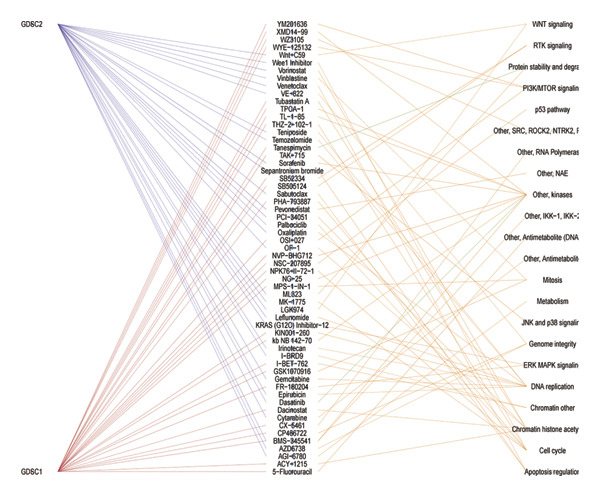
(c)

**Figure:**
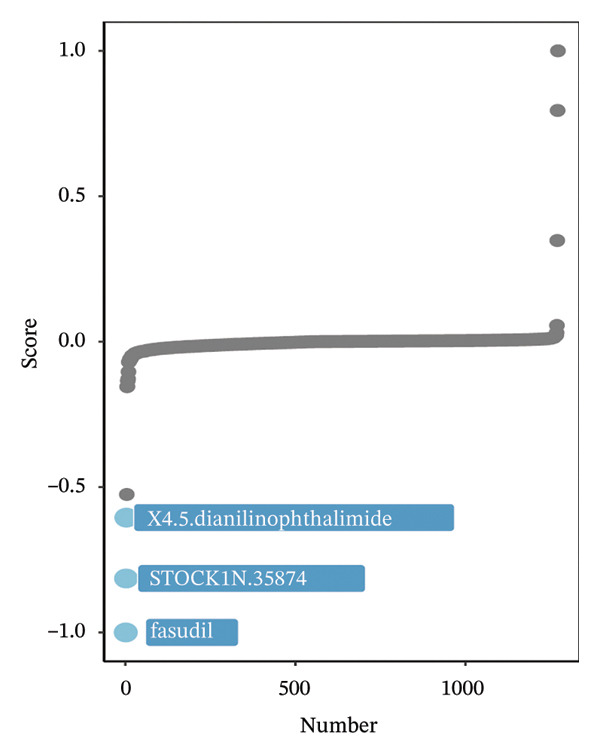
(d)

In parallel, a cMAP analysis using the XSum algorithm prioritized compounds predicted to reverse the BDKRB1‐associated transcriptional signature (300 genes; 150 up‐ and 150 downregulated in BDKRB1‐high tumors). Fasudil (HA‐1077), a ROCK inhibitor, emerged as a representative top‐ranked candidate with a strongly negative connectivity score, indicating an inverse transcriptional relationship relative to the BDKRB1‐associated signature among 1288 perturbational profiles (Figure 8(d)). Additional candidates (e.g., X4.5.dianliinophthalmide and STOCK1N.35874) showed less extreme inverse connectivity. Together, these analyses nominate testable therapeutic hypotheses—both drug sensitivity associations and connectivity‐based candidates—that warrant follow‐up validation in ovarian cancer–relevant experimental systems.

## 4. Discussion

Ovarian cancer remains one of the most challenging malignancies in women’s health, characterized by high mortality rates primarily due to late‐stage diagnosis and frequent disease recurrence. The aggressive nature of this disease, combined with limited early detection methods and the rapid development of therapy resistance, underscores the urgent need for a deeper understanding of its molecular drivers. Our study provides comprehensive evidence that BDKRB1 plays a multifaceted role in ovarian cancer pathogenesis, influencing prognosis, genomic stability, TME composition, and therapeutic response. Importantly, the integrated evidence presented here is primarily observational and association‐based and thus should be interpreted as defining a reproducible tumor ecosystem state linked to BDKRB1 rather than establishing causality. The findings presented here not only establish BDKRB1 as a significant biomarker but also reveal its potential as a therapeutic target, offering new insights into the complex biology of this devastating disease.

The prognostic significance of BDKRB1 in ovarian cancer is particularly striking. Our analysis of multiple independent cohorts consistently demonstrated that high BDKRB1 expression correlates with worse OS and DSS. This association was robust across different analytical methods, suggesting that BDKRB1 overexpression is a reliable indicator of aggressive disease. The observation that BDKRB1’s prognostic value was most pronounced for OS rather than PFI raises interesting questions about its role in long‐term disease outcomes versus initial treatment response. One plausible interpretation is that BDKRB1 marks longer–horizon tumor ecosystem features (e.g., chronic stromal remodeling and sustained immune dysfunction) that accumulate impact on OS/DSS, whereas PFI/DFI may be more sensitive to short‐term treatment and perioperative factors. One possible explanation is that BDKRB1 may be more involved in fundamental processes like metastatic dissemination or therapy resistance development rather than initial tumor growth or short‐term treatment sensitivity. The genomic instability associated with BDKRB1‐high tumors, as evidenced by increased fractions of the genome altered, gained, and lost, provides a potential mechanism for this aggressive behavior. Chromosomal instability (CIN) is a well‐known hallmark of advanced cancers, often associated with increased tumor heterogeneity and adaptability. CIN often results from errors in chromosome segregation during mitosis, leading to aneuploidy and other chromosomal aberrations. This instability is especially prevalent in various malignancies, including breast cancer and lung cancer, where it has been linked with worse prognosis and increased rates of metastasis [22]. Another crucial aspect of CIN is its relationship with tumor cell plasticity, wherein variable karyotypes are generated that can propagate traits favoring survival and drug resistance [23]. Furthermore, research by Andor et al. highlighted a paradigm where CNVs, rather than single mutations, are pivotal in establishing and maintaining intratumor heterogeneity, further reinforcing that CIN is integral to cancer evolution and adaptability [24]. In our data, BDKRB1 copy number and mRNA expression were significantly correlated but with a modest effect size, supporting the interpretation that genomic amplification may contribute to BDKRB1 upregulation while additional nongenetic regulatory mechanisms are also likely involved. Our finding that BDKRB1 copy number alterations correlate with its mRNA expression suggests that genomic amplification may be a key driver of BDKRB1 overexpression in ovarian cancer, although the correlation strength implies only partial dosage dependence rather than a sole driver, creating a potential positive feedback loop that maintains its oncogenic activity.

The impact of BDKRB1 on the TME represents another critical aspect of our findings. The immune landscape of BDKRB1‐high tumors presents a complex picture, with simultaneous upregulation of both immunosuppressive and immunostimulatory markers. Rather than representing a contradiction, this pattern is consistent with an “inflamed but ineffective” immune state, in which inflammatory transcriptional programs coexist with suppressive or spatially constrained immune function. This apparent paradox may reflect an attempt by the immune system to mount an antitumor response that is ultimately thwarted by BDKRB1‐driven immunosuppressive mechanisms. The enrichment of CAFs in BDKRB1‐high tumors is particularly noteworthy, as CAFs are increasingly recognized as key players in creating a protumorigenic microenvironment, influencing tumor growth and progression through various paracrine signaling pathways [25, 26]. Our single‐cell RNA sequencing data, which consistently identified fibroblasts as the predominant expressors of BDKRB1 across multiple cohorts, strongly suggests that bulk BDKRB1‐associated immune and pathway signals may be stromal‐centered rather than purely tumor cell intrinsic and that BDKRB1‐associated signaling in these stromal cells could contribute to shaping the TME. This finding aligns with growing evidence that stromal–tumor interactions are crucial in ovarian cancer progression and therapy resistance [27, 28]. The primary barrier to effective immunotherapy in ovarian cancer lies in its immunosuppressive TME. Ovarian tumors are characterized by the accumulation of regulatory T cells (Tregs) and myeloid‐derived suppressor cells (MDSCs), which inhibit the activation and function of effector T cells [29]. This immunosuppression is further exacerbated by elevated levels of immunosuppressive cytokines such as IL‐10 and TGF‐β, produced by these cell populations. The observed exclusion of CD8+ T cells in BDKRB1‐high tumors further supports the notion that BDKRB1 creates an immune–hostile environment, potentially explaining the limited success of immunotherapies in ovarian cancer to date. Notably, we explicitly distinguish deconvolution‐derived immune cell abundance estimates from gene expression–based immune activity signatures, and we interpret immune infiltration results by emphasizing concordant directional trends across multiple algorithms rather than absolute values from any single method.

At the molecular level, BDKRB1 appears to function as a signaling hub, influencing multiple oncogenic pathways simultaneously. At the transcriptome level, pathway‐inference analyses (GSEA/GSVA/PROGENy) indicate that BDKRB1‐high tumors are associated with broad pathway‐state differences; however, these results reflect transcriptional footprints and do not, by themselves, establish direct upstream signaling activation or causality. The widespread activation of pathways such as EGFR, MAPK, and PI3K in BDKRB1‐high tumors suggests that BDKRB1 overexpression creates a permissive environment for cancer cell survival and proliferation. The concurrent suppression of DNA damage response pathways provides a plausible explanation for the genomic instability observed in these tumors, as impaired DNA repair mechanisms would naturally lead to increased mutation accumulation. The genomic landscape of ovarian cancer is frequently marked by mutations in genes regulating key DDR pathways. Mutations and alterations in specific signaling pathways, particularly those linked to the PI3K/Akt/mTOR cascade, play a significant role in the genomic instability observed in many ovarian tumors [30]. The dysregulation of these pathways enhances cell proliferation and diminishes the effective repair of DNA damage, establishing a cycle that promotes tumorigenesis. The metabolic reprogramming associated with BDKRB1 overexpression, particularly the upregulation of glycan and lipid biosynthesis pathways, may represent another layer of BDKRB1’s oncogenic activity. More conservatively, these metabolic shifts may represent coordinated features of the BDKRB1‐high tumor ecosystem state, potentially linked to stromal remodeling and inflammatory signaling. These metabolic changes could contribute to membrane receptor stabilization, energy production for rapid proliferation, or generation of signaling molecules that further reinforce oncogenic pathways.

The therapeutic implications of our findings are particularly exciting. The identification of BDKRB1 as a potential target opens new avenues for treatment development. The negative correlation between BDKRB1 expression and sensitivity to certain kinase inhibitors suggests that BDKRB1‐high tumors may have unique vulnerabilities that can be exploited therapeutically. However, because these drug sensitivity associations are derived from in vitro reference datasets and computational inference, they should be viewed as exploratory and hypothesis‐generating rather than clinically actionable. The strong performance of fasudil in our cMAP analysis is especially intriguing, as this ROCK inhibitor has already shown promise in other cancer types and could potentially be repurposed for BDKRB1‐high ovarian cancers. In this context, fasudil should be interpreted as a top‐ranked connectivity‐based candidate predicted to inversely relate to the BDKRB1‐associated transcriptional program, requiring experimental validation in ovarian cancer–relevant models. Fasudil, a Rho kinase inhibitor, has emerged as a promising therapeutic agent in cancer treatment due to its multifaceted mechanisms of action that involve inhibition of cell proliferation, migration, invasion, and enhanced apoptosis across various cancer types. Initially approved for treating cerebral vasospasm in Japan, fasudil’s safety profile has already been established, allowing for its potential repositioning in oncology with minimal risk of severe adverse effects [31, 32]. The fact that BDKRB1 expression correlates with sensitivity to HSP90 inhibitors provides another potential therapeutic angle, as HSP90 is known to stabilize numerous oncoproteins. HSP90 functions by ensuring that its client proteins maintain proper conformational states, thus enabling their activity and stability under physiological conditions. This is particularly relevant in cancer, where mutations or aberrations in client proteins such as BRAF, AKT, and HER2 lead to oncogenic signaling (heat shock protein 90 inhibition: rationale and clinical potential). Inhibition of HSP90 results in the degradation of these client proteins via the ubiquitin–proteasome pathway, ultimately impairing the cancer cell’s ability to grow and survive [33]. Collectively, these results support BDKRB1 as a candidate biomarker for patient stratification for generating testable therapeutic hypotheses, while prospective validation is required before any predictive biomarker claims. These findings collectively suggest that BDKRB1 could serve not only as a prognostic marker but also as a predictive biomarker for specific treatment approaches.

In conclusion, our comprehensive multiomics analysis establishes BDKRB1 as a central player in ovarian cancer pathogenesis, influencing disease progression through multiple interconnected mechanisms. More precisely, our data position BDKRB1 as a stromal‐enriched marker that is reproducibly associated with copy number–defined genomic instability and an inflamed but functionally constrained microenvironment in ovarian cancer. From its prognostic significance to its role in shaping the TME and modulating therapeutic response, BDKRB1 emerges as a molecule of considerable clinical interest. These findings not only advance our understanding of ovarian cancer biology but also provide a foundation for developing new stratification strategies and targeted therapies. As we continue to unravel the complexities of this disease, BDKRB1 represents a promising focus for future research efforts aimed at improving outcomes for ovarian cancer patients. Future work should prioritize mechanistic validation in models that preserve stromal–immune context and prospective evaluation in clinically annotated cohorts to define the most appropriate clinical use case for BDKRB1. The integration of BDKRB1 assessment into clinical practice could potentially enable more personalized treatment approaches, while further investigation of its molecular functions may reveal additional therapeutic opportunities. Ultimately, this work contributes to the growing body of knowledge that is gradually transforming our ability to combat this challenging disease.

While our study provides substantial new insights, several limitations should be acknowledged. The retrospective nature of our analyses means that causal relationships cannot be definitively established without functional validation. In addition, immune deconvolution and pathway inference analyses rely on computational modeling and thus should be interpreted as indicative of ecosystem‐level associations rather than direct mechanistic measurements. The complexity of BDKRB1’s effects across different cellular compartments further suggests context‐dependent functions within the TME. Finally, pharmacogenomic associations and connectivity mapping results are exploratory and require validation in ovarian cancer–relevant experimental systems before clinical translation.

## 5. Conclusion

In summary, this multiomics study highlights BDKRB1 as a reproducible marker associated with genomic instability and a stromal‐centered immune context in ovarian cancer. The integration of bulk and single‐cell analyses provides a systems‐level perspective on how inflammatory and stromal programs converge in BDKRB1‐high tumors. These results offer a foundation for future validation studies and potential biomarker‐oriented stratification strategies.

## Author Contributions

D.P. conceived and designed the study. H.C. and X.W. analyzed data. D.P. collected data. D.P. and X.W. wrote. S.H. helped with the final revision of this manuscript.

## Funding

This research received no specific funding from any funding agency in the public, commercial, or not‐for‐profit sectors.

## Disclosure

All authors reviewed and approved the final manuscript.

## Ethics Statement

The authors have nothing to report.

## Conflicts of Interest

The authors declare no conflicts of interest.

## Data Availability

The datasets used and/or analyzed during the current study are available from the corresponding author on reasonable request.
